# Blessing in disguise: A loss of miR159 makes plant drought tolerant and ABA sensitive

**DOI:** 10.1111/ppl.13763

**Published:** 2022-09-12

**Authors:** Manish Tiwari

**Affiliations:** ^1^ Department of Bacteriology University of Wisconsin‐Madison Madison Wisconsin USA

Abiotic and biotic environmental stimuli drove the evolution of primitive living organisms. An important abiotic factor, water availability, continuously steers the plant evolution over the years. Drought stress prevents plants from realizing their full genetic potential and is a significant hurdle in sustainable agriculture (Rane et al., 2022). Several reports on climate change predict the loss of agricultural yields due to drought stress (Corwin, 2021). Understanding the cellular dynamics and underlying molecular mechanism of drought tolerance can fulfill the goal of engineering plants with increased productivity under stress conditions. ABA signaling directly regulates stomatal opening and transpiration and is the prime candidate responsible for deciding the cellular fate under stress conditions. Moreover, ABA sensitivity is tightly associated with plant drought tolerance and seed dormancy (Mega et al., 2019; Tuan et al., 2018). Therefore, the role of ABA signaling in drought stress during seed germination can be crucial for developing stress‐resilient crops.

In this issue of *Physiologia Plantarum*, Jiang et al. (2022) established a signaling cascade involving miR159, MYB33, and ABA‐responsive basic leucine zipper transcription factor ABI5 regulating Arabidopsis seed germination under drought stress. To investigate the relationship between miR159 and its target MYB33, expression analysis was conducted, which showed that miR159 was decreased while MYB33 expression was increased during drought stress. *In‐planta* functional characterization of miR159 during drought was performed using the loss of function mutant lines. The miR159ab mutant lines displayed a higher survival rate than Col‐0 under drought stress reflecting that downregulating miR159 expression makes plant drought tolerant. Intriguingly the miR159ab mutant lines exhibited smaller stomata size, higher stomatal density, and significantly lower number of open stomata under drought conditions suggesting mutants endured drought conditions by decreasing the number of open stomata, thereby preserving water loss by transpiration. Further seed germination assay of miR159ab mutants in the presence of ABA revealed a decreased seed germination compared to control. In contrast, both mutants and control plants showed similar seed germination rates without ABA suggesting that miR159ab was hypersensitive toward ABA. In the presence of ABA, only single mutant miR159ab seeds showed reduced germination, whereas single mutants (▲*myb33* and ▲*abi5‐2*), double mutant (▲*myb33▲abi5‐2*), and triple mutants (▲miR159ab▲myb33 and ▲mir159ab▲abi5‐2) displayed either increased germination or same as Col‐0.

**FIGURE 1:**
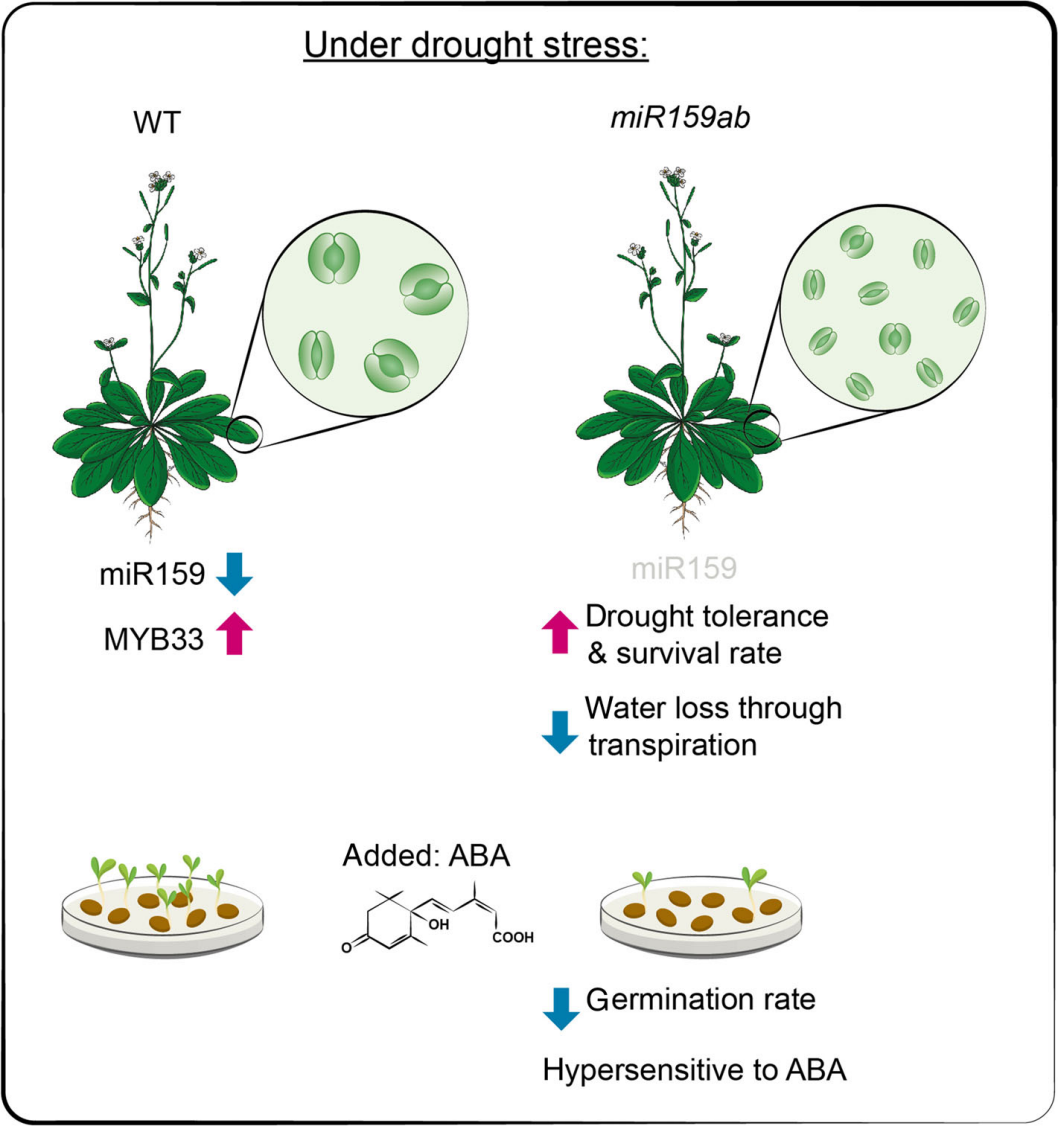
A model depicts the role of miR159 in stress tolerance and seed germination under drought. miR159 levels get reduced under drought stress in wild‐type Arabidopsis plants leading to higher expression of corresponding target MYB33. Similarly, *miR159ab* knockdown mutants with lower expression of miR159 and suppression of posttranscriptional silencing of MYB33 show stomatal closure, reduced water loss through transpiration, drought stress tolerance, and a higher survival rate. The low level of miR159 also leads to hypersensitivity to ABA and a reduction in seed germination during drought stress.

Through seed germination assays in different mutants, authors clearly showed that knocking down miR159 negatively regulates seed germination, whereas knocking down MYB33 or ABI5 gene can increase seed germination (Figure 1). The findings indicate a genetic interaction among the miR159, MYB33, and ABI5. This led the authors to conclude a regulatory network in which miR159 regulates MYB33, which regulates ABI5 and thus the drought stress response during seed germination.

Upon facing drought stress, plants need to degrade positive regulators of ABA signaling such as ABI5 to reboot the developmental program culminating the juvenile development to finish their life cycle. It has been established earlier that drought stress regulates vegetative phase transition by miR159 level and subsequent target MYB33. Downstream MYB33, ABI5, is activated, which orchestrates ABA signaling (Guo et al., 2021). However, the role of drought‐induced ABA signaling during seed germination remained elusive. In conclusion, Jiang et al. presented a regulatory module for seed germination in which they used exogenous ABA as a signaling cue to mimic drought stress effects. Through mutant analysis of miR159, MYB33, and ABI5, they found the functional signaling cascade during seed germination under drought stress, which can be genetically engineered for better yield under limiting water availability.
